# Draft Genome Sequence of the Fish Pathogen *Flavobacterium columnare* Strain CSF-298-10

**DOI:** 10.1128/genomeA.00173-17

**Published:** 2017-04-13

**Authors:** Jason P. Evenhuis, Scott E. LaPatra, Joerg Graf

**Affiliations:** a U.S. Department of Agriculture, National Center for Cool and Cold Water Aquaculture, Agriculture Research Service, Kearneysville, West Virginia, USA; b Clear Springs Foods Inc., Buhl, Idaho, USA; c Department of Molecular and Cell Biology, University of Connecticut, Storrs, Connecticut, USA

## Abstract

We announce here the draft genome assembly of *Flavobacterium columnare* CSF-298-10, a strain isolated from an outbreak of columnaris disease at a commercial trout farm in Hagerman Valley, Idaho, USA. The complete genome consists of 13 contigs totaling 3,284,579 bp, with an average G+C content of 31.5% and 2,933 predicted coding genes.

## GENOME ANNOUNCEMENT

Columnaris disease has caused losses in multiple aquaculture species (1, 2) and is an emerging bacterial problem in the rainbow trout industry (3, 4). The ecological agent *Flavobacterium columnare* is a Gram-negative, yellow-pigmented, rod-shaped bacterium that has wide genomic heterogeneity and host virulence variations (5 – 8). *Flavobacterium columnare* isolates are classified in one to five different genomovar types, and all isolates causing disease outbreaks in the rainbow trout industry are genomovar type I. The CSF-298-10 strain is a genomovar I strain that was isolated in 2010 from the gill tissue of a diseased rainbow trout (4) in the Snake River valley of Idaho, USA. This strain is currently being used to initiate a breeding program to improve innate survivability against columnaris disease (9).

*F. columnare* CSF-298-10 was cultured in tryptone yeast extract salt broth medium at 30°C and 150 rpm (Innova44 incubator, New Brunswick Scientific). Genomic DNA was isolated from 1.5 mL of overnight culture using the MasterPure Gram-positive DNA purification kit (Epicentre), and sent away for Illumina HiSeq sequencing at Arizona Research Labs (Tucson, AZ, USA). Quality filtering and trimming were performed using bcl2fastq conversion software version 1.8.2 and Trimmomatic version 0.30 with parameters TRAILING:3. PacBio sequencing was performed at the Keck DNA Sequencing Center at Yale University. PacBio reads and Illumina reads were assembled using SPAdes version 3.9.1. The annotation was done using the NCBI Prokaryotic Genome Annotation Pipeline.

The draft genome has a total size of 3,284,579 bp represented in 13 contigs with an *N*_50_ value of 378,142 bp. The average G+C content is 31.5%, which is consistent with the other *F. columnare* genomovar I strains previously sequenced, namely, ATCC 49512 (31.5%) (10) and Pf1 (31.58%) (11), and is slightly higher than the G+C content of the previously sequenced genomovar II strains C#2 (30.97%) (12) and 94-081 (30.8%) (13). A total of 2,911 genes encoding 2,836 open reading frames representing 318 different subsystems were identified. The genome contained one complete ribosomal operon, 69 tRNAs, three ncRNAs, and three CRISPR arrays. CRISPR arrays were also reported to be present in *F. columnare* strains 94-081 and C#2. There were several interesting genes present in the genome that could be related to virulence, including five that were similar to internalin, a suite of genes comprising a conjugative transposon, siderophore biosynthetic genes, and four catalases or peroxidases.

The CSF-298-10 *F. columnare* strain is currently being used to breed a line of rainbow trout with improved survivability against CSF-298-10 infections. This line of trout will be useful to reduce losses associated with columnaris disease and to compare other virulent strains of *F. columnare*. The availability of the CSF-298-10 genome will allow genome-wide comparisons of virulence factors and help in elucidating the underlying genetic requirements for virulence.

### Accession number(s).

This whole-genome shotgun project has been deposited at DDBJ/EMBL/GenBank under the accession number MUAW00000000. The version described in this paper is the first version, MUAW01000000.
